# Cytomegalovirus pneumonia with intermittent pulmonary hemorrhage leading to asphyxia death: a case report and literature review

**DOI:** 10.1186/s12985-024-02399-7

**Published:** 2024-06-05

**Authors:** Chenguang Yang, Qi Ge, Xiaochuan Huo, Chang Ge

**Affiliations:** 1 Liyuan Hospital, Tongji Medical College, Huazhong University of Science and Technology, Wuhan, 430077 China; 2 Department of Forensic Medicine, Tongji Medical College, Huazhong University of Science and Technology, Wuhan, 430030 China; 3 Sanquan College of Xinxiang Medical University, Xinxiang, 453003 China

**Keywords:** Neonatal, Intermittent pulmonary hemorrhage, Autopsy, CMV pneumonia, Interstitial pneumonia, Asphyxia

## Abstract

Neonatal pulmonary hemorrhage is a late manifestation of various diseases. Premature delivery and low body weight are frequently observed as high-risk factors, characterized by acute onset, rapid progression, and high mortality rates. Pulmonary hemorrhage caused by cytomegalovirus infection in newborns with normal immune function is a rare occurrence. This case report focuses on a term neonate with normal birth weight who presented solely with nasal obstruction shortly after birth. However, 4 days after birth, the newborn experienced a sudden onset of blood gushing from both the mouth and nasal cavity. The patient was diagnosed with gastrointestinal bleeding, neonatal pneumonia and neonatal lung consolidation. And he was discharged after ten days of symptomatic treatment. However, upon returning home, the patient experienced a sudden onset of bleeding from the mouth and nose, leading to his untimely demise. Subsequent autopsy revealed the presence of pulmonary hemorrhage in newborn, which presented as interstitial pneumonia. The cause of pulmonary hemorrhage is cytomegalovirus infection. This case emphasizes the importance of pediatricians enhancing their skills in differentiating pulmonary hemorrhage, especially from cytomegalovirus pneumonia.

## Introduction

The incidence of pulmonary hemorrhage in live births ranges from 1‰ to 12‰, with a mortality rate as high as 50 − 80% [1]. Neonatal pulmonary hemorrhage can be caused by pulmonary dysplasia, coagulation dysfunction, infection, hypoxia, and other factors, often affecting more than two lobes [2]. The etiology of full-term neonatal pulmonary hemorrhage is complex, and the clinical manifestations are atypical. X-ray examination lacks specificity in diagnosing pulmonary hemorrhage. When pulmonary hemorrhage occurs, blood gushes from the mouth and nose, which can sometimes lead to misdiagnosis as upper gastrointestinal bleeding.

### Case

#### Clinical data

A 30-year-old female at 38 weeks of gestation, with one live birth and one induced abortion prior to this pregnancy, underwent a prenatal examination which showed no abnormalities except for a positive result for group B streptococcus. The pregnant woman gave birth to a baby boy vaginally. The newborn exhibited slight nasal congestion and 1-min Apgar score of 9 and 5-min Apgar score of 10. The neonatal hearing and behavioral responses are normal. Four days later, he suddenly experienced hematemesis, with blood spilling from the mouth and nasal cavity. Upon admission, the physical examination revealed a body temperature of 36.5 ℃, a heart rate of 138 beats per minute, a respiratory rate of 50 breaths per minute, and a blood oxygen saturation level of 95%. Laboratory examination results revealed a white blood cell count of 12.37 × 10^9^/L, a red blood cell count of 4.58 × 10^12^/L, a hemoglobin concentration of 159 g/L, a hematocrit of 0.469, a platelet count within normal range at 255 × 10^9^/L, a lymphocyte percentage of 13.7%, and a neutrophil percentage of 75.3%. Coagulation function tests showed an elevated prothrombin time of 15.8 s, an elevated activated partial thromboplastin time of 43.3 s, and an elevated D-dimer level of 0.51 ug/ml, and normal fibrinogen levels. Liver function tests indicated a normal ALT of 12U/L and a normal AST of 36U/L. Procalcitonin levels were within the normal range at 0.38ng/L, high-sensitivity C-reactive protein level was also within the normal range at 0.73 mg/L, and the human serum amyloid protein level was normal, measuring less than 1 mg/L. Blood bacterial culture results were negative. Chest X-ray findings revealed thickened and blurred lung texture, along with patchy and fuzzy shadows in both lungs, some of which were consolidated. The trachea was unobstructed. Given the observation of blood gushing from the mouth and nose, the initial diagnosis was upper gastrointestinal bleeding, neonatal pneumonia and neonatal lung consolidation. The patient received treatment including fasting, gastrointestinal decompression, vitamin K for hemostasis, penicillin, and cefotaxime for anti-infection. The child no longer vomited blood after treatment.

The child was discharged from the hospital ten days later without experiencing hematemesis, fever, or dyspnea. The laboratory examination results showed a Cytomegalovirus IgG antibody level of > 1000.00 (AU/ml), indicating a high presence of antibody. This prompted the doctor to consider the possibility that the child’s mother had a Cytomegalovirus infection. However, at approximately 23 o’clock on the same day, the child experienced hematemesis again, with blood gushing from the mouth and nose. Despite rescue efforts, the child unfortunately did not survive.

### Postmortem examination

**Fig. 1:**
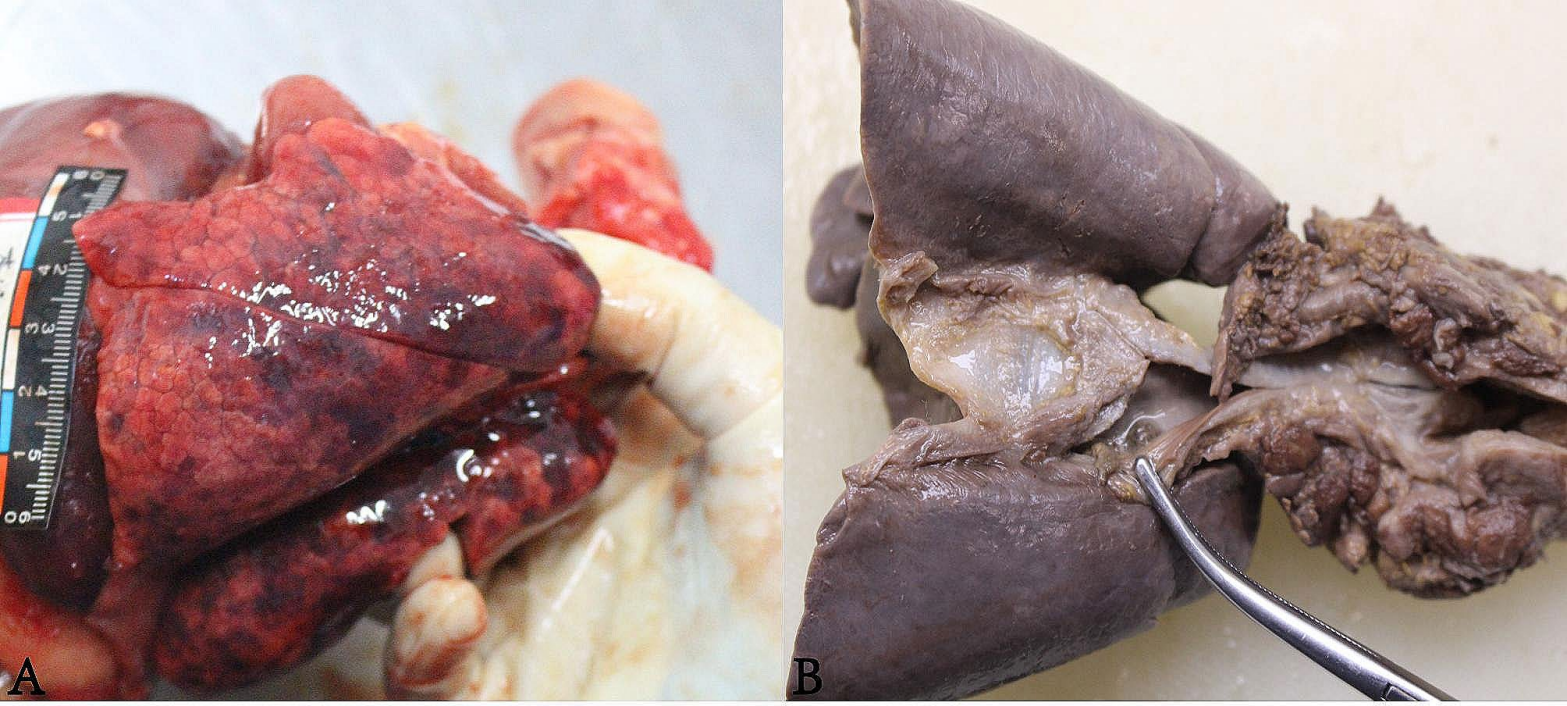
Gross examination. (**A**): Lamellar hemorrhage. (**B**): Clot in bronchial lumen

The measurements of the infant are as follows: weight − 3900 g, height − 51 cm, chest circumference − 36 cm, abdominal circumference − 31 cm, and head circumference − 36 cm. During examination, there were no bleeding spots observed in the bulbar conjunctiva and palpebral conjunctiva, but cyanosis was observed in the facial and labial mucosa. The heart weighed 30.5 g, and the cardiac chambers were dissected in the direction of blood flow. The thickness of the left and right ventricular walls was measured to be 0.3 cm and 0.2 cm, respectively. The ductus arteriosus remained open and there were no signs of congenital heart disease. The left lung weighed 41.5 g, while the right lung weighed 51.5 g. The surface of the lungs showed multiple patchy hemorrhages (Fig. 1A). Additionally, the lungs appeared solid and the bronchial lumens were obstructed by blood clots on both sides (Fig. 1B).

## Pathological findings

**Fig. 2:**
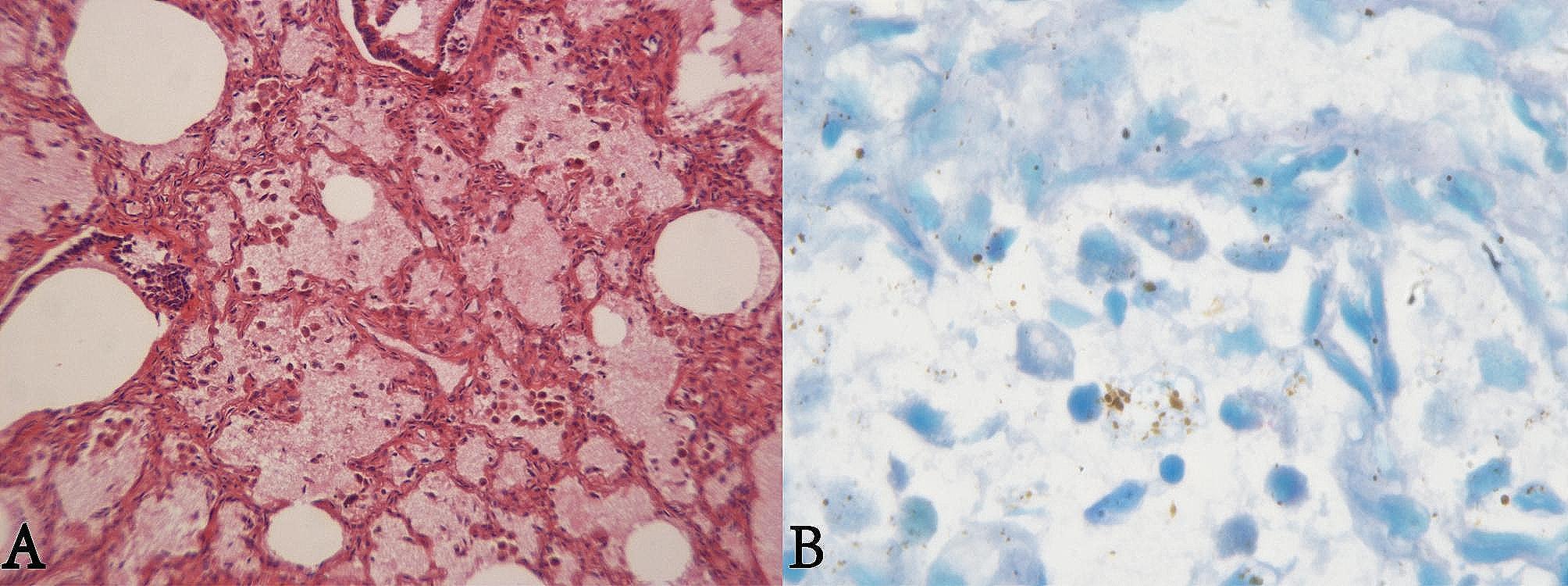
Microscopic observation. (**A**): Red blood cells in alveolar and bronchiolar lumen (HE×100). (**B**): Vacuoles in macrophages and alveolar epithelial cells (Giemsa×1000)

Under the microscope, it was observed that some alveolar cavities were dilated and inflated, pulmonary bronchioles were well developed, and some alveolar septa were widened. Additionally, red blood cells are present in extensive bronchiolar and alveolar spaces, along with scattered macrophages in the alveolar spaces(Fig. 2A). The liver weighed 115 g and microscopically, the liver plate and liver cord were well developed. No ulcers were found in the esophageal and gastric walls, and there was no accumulation of blood in the intestinal tract. Giemsa staining of lung tissue revealed vacuolization of the nuclei of alveolar epithelial cells and macrophages in the alveolar space (Fig. 2B).

**Fig. 3:**
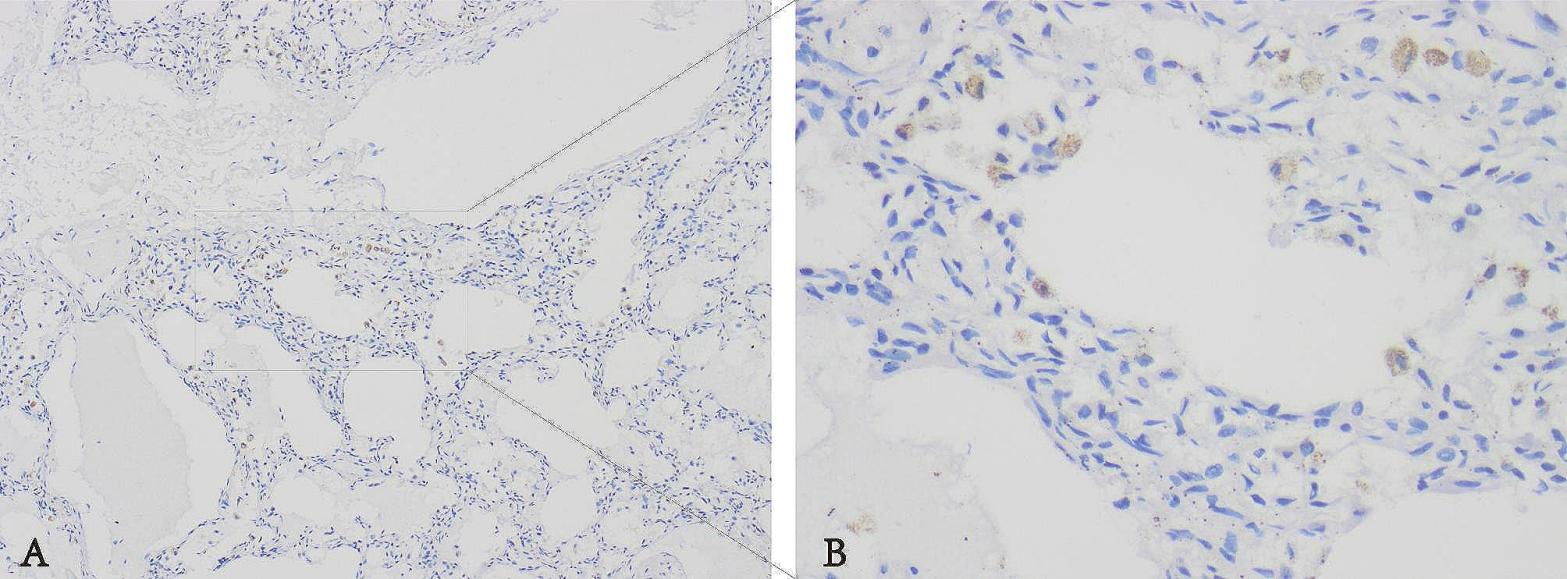
The expression of CMV antigen in the lung (**A**)×100, (**B**) is the magnifications of A respectively, ×400)

The expression of CMV antigen was examined using immunohistochemistry with a polyclonal mouse anti-CMV antibody. CMV antigen was expressed in alveolar epithelium and intra-alveolar macrophages (See Fig. 3).

## Expert opinion

The cause of death in the child was determined to be respiratory tract obstruction and asphyxia resulting from pulmonary hemorrhage associated with interstitial pneumonia.

## Discussion

Research has demonstrated that Cytomegalovirus has the ability to pass through the placental barrier, leading to congenital infection in the, fetus. Some children affected by this infection may exhibit symptoms such as mental retardation, neuromuscular movement disorders, deafness, and chorioretinitis [3]. Perinatal infections can occur due to exposure to infected maternal body fluids during birth. It is noted that most newborns infected with CMV are either asymptomatic or experience mild symptom. Additionally, CMV pneumonia is rare in full-term newborns, with CMV pneumonia resulting from congenital infection being even more uncommon [4].

Choi J et al. reported a case study of a 2-month-old female infant diagnosed with cytomegalovirus infection [5]. The mother of the baby girl underwent routine prenatal examination and delivered the baby naturally at full term. The infant had average weight and no metabolic or genetic diseases. Laboratory examination revealed a white blood cell count of 23180cells/mm^3^, with 62% neutrophils, 30% lymphocytes, and 8% monocytes. The C-reactive protein level was 0.1 mg/dl, ESR was 16 mm/hr, hemoglobin level was 9.3 g/dl, and platelet count was 461,000/mm^3^. Chest X-ray revealed diffuse bilateral haziness in both lung fields. PCR detection of blood and alveolar lavage fluid confirmed the presence of Cytomegalovirus, leading to the administration of ganciclovir for treatment. The infant’s condition stabilized, with subsequent negative cytomegalovirus test results, and was discharged after achieving normal oxygen saturation levels during spontaneous breathing.

### Pathogenesis of cytomegalovirus

Human Cytomegalovirus (HCMV) belongs to the β Herpesvirus family and has a DNA-based genetic material. Its genome consists of a large linear double-stranded DNA with approximately 100 to 200 open reading frames (ORFs) [6]. The genome is located at the center and is surrounded by an icosahedral capsid, complex tegument, and glycoprotein-modified envelope. The entry of the virus into host cells involves two stages: fusion and interference. In the fusion stage, the virus fuses with the host cell membrane using various envelope proteins that resemble the host cell membrane, including receptor protein (GD), activator (the gh/gl complex), and fusion protein (GB), thereby binding the virus to the cell. In the interference stage, the viral membrane fuses with the host’s plasma membrane or endocytic membrane through the virus’s fusion protein, thereby interfering with the host membrane [7]. Upon entering the host cell, the nucleocapsid enters the nucleus, undergoes cleavage, and most ORFs are expressed to produce subviruses.

### CMV reactivations with variable frequency

Viral glycoproteins and nucleic acids, known as pathogen-associated molecular patterns (PAMPs), can be recognized by the human immune system. This recognition activates the innate immune response and triggers interferon (IFN) signals [8]. Immediate-early genes in ORFs play a protective role against the influence of innate immunity [9]. Additionally, CMV has developed various effectors that down-regulate the sensing and signaling of IFN-induced pathways, as well as the expression and function of interferon-stimulated genes (ISGs). CMV can also disrupt the apoptosis process caused by endoplasmic reticulum (ER) stress and DNA damage [10]. By inhibiting the IFN response, CMV can enter a period of incubation after primary infection [11].

During latent infection, CMV produces immunosuppressive cytokines such as IL-10 and TGF-β, which can inhibit the cytotoxicity of T lymphocytes and maintain dormancy in host cells [12, 13]. Furthermore, latent CMV infection can recruit activated CD4 + T lymphocytes, leading to virus reactivation [14]. Once activated, the virus replicates, surpassing the threshold of host immunity, and resulting in varying degrees of clinical symptoms [15]. Due to the correlation between viral load and the body’s immune response, CMV-infected pneumonia frequently manifests with intermittent symptoms.

### Cytomegalovirus pneumonia and pulmonary hemorrhage

Neonatal cytomegalovirus pneumonia typically results from perinatal infection, with the virus directly entering the lungs through the respiratory tract [16]. Generally, Cytomegalovirus primarily infects epithelial cells, endothelial cells, and fibroblasts. However, in lung tissue, the main targets are alveolar epithelium, giant cells in the alveolar cavity, and interstitial cells [17]. Upon infecting type II alveolar epithelial cells, Cytomegalovirus reduces surfactant production [18]. Severe cases may experience hypoxemia and increased vascular permeability, potentially leading to pulmonary hemorrhage [19].

### Early diagnosis of cytomegalovirus pneumonia with pulmonary hemorrhage

Detecting pulmonary hemorrhage on chest X-rays can be challenging due to underlying diseases. Serial analysis of X-rays is crucial in diagnosing neonatal pulmonary hemorrhage [20]. Ultrasound, being less harmful and more sensitive than X-rays, has emerged as a reliable tool for diagnosing pneumonia, pneumothorax, and respiratory distress syndrome [21]. It is particularly useful for dynamic monitoring of the, condition and aids in early detection of pulmonary hemorrhage [22].

The gold standard for diagnosing congenital CMV infection is isolating the virus from human fibroblasts within 2 weeks of birth. For CMV-infected pneumonia, the virus can be isolated from target cells such as alveolar epithelial cells, interstitial cells, or alveolar macrophages.In clinical practice, the diagnosis of CMV pneumonia is primarily based on symptom evaluation, imaging results, and positive CMV PCR tests in blood, urine, and BAL fluid. Additionally, IgM antibodies can be found in 20–70% of newborns with the infection [23]. Detecting CMV-IgM antibodies in the blood within two weeks of birth is crucial for distinguishing between congenital and perinatal infections. Timely diagnosis aids in developing effective treatment strategies, ultimately lowering the incidence of severe pneumonia and pulmonary hemorrhage [24].

This case study presents a rare occurrence of pneumonia induced by cytomegalovirus infection in a child, complicated by intermittent pulmonary hemorrhage and resulting in death due to asphyxiation. The infant displayed noticeable clinical symptoms on the fourth day post-birth and succumbed on the 14th day post-birth. Postmortem examination of lung tissue confirmed the presence of cytomegalovirus, meeting the diagnostic criteria for congenital infection. The significantly elevated cytomegalovirus IgG antibody titer in laboratory tests, along with the characteristic intermittent symptomatology following cytomegalovirus infection, supported the diagnosis of congenital cytomegalovirus infection. The child experienced concurrent pneumonia and pulmonary hemorrhage, with the pneumonia showing improvement following anti-infective treatment, while the pulmonary hemorrhage persisted and ultimately led to airway obstruction and death by suffocation.

## Conclusion

This unique case serves to highlight the potential of CMV pneumonia to cause intermittent pulmonary bleeding. When clinical suspicion arises of neonatal cytomegalovirus pneumonia, it is essential to conduct thorough etiological examinations and remain vigilant for the occurrence of pulmonary hemorrhage. Early detection and prompt initiation of appropriate treatment measures can significantly benefit the child’s recovery.

## Data Availability

The datasets used and/or analyzed during the current study are available from the corresponding author on reasonable request.
